# Potential Role of MicroRNAs in the Regulation of Antiviral Responses to Influenza Infection

**DOI:** 10.3389/fimmu.2018.01541

**Published:** 2018-07-04

**Authors:** Thi Hiep Nguyen, Xiaoming Liu, Zhen Zhong Su, Alan Chen-Yu Hsu, Paul S. Foster, Ming Yang

**Affiliations:** ^1^Priority Research Centre for Healthy Lungs, The University of Newcastle, Callaghan, NSW, Australia; ^2^Faculty of Health and Medicine, School of Biomedical Sciences and Pharmacy, The University of Newcastle, Callaghan, NSW, Australia; ^3^Department of Respiratory Medicine, The Second Hospital, Jilin University, ChangChun, China; ^4^Faculty of Health and Medicine, School of Medicine and Public Health, The University of Newcastle, Callaghan, NSW, Australia

**Keywords:** microRNA, immune responses, influenza virus, infection, inflammation

## Abstract

Influenza is a major health burden worldwide and is caused by influenza viruses that are enveloped and negative stranded RNA viruses. Little progress has been achieved in targeted intervention, either at a population level or at an individual level (to treat the cause), due to the toxicity of drugs and ineffective vaccines against influenza viruses. MicroRNAs (miRNAs) are small non-coding RNAs that play critical roles in gene expression, cell differentiation, and tissue development and have been shown to silence viral replication in a sequence-specific manner. Investigation of these small endogenous nucleotides may lead to new therapeutics against influenza virus infection. Here, we describe our current understanding of the role of miRNAs in host defense response against influenza virus, as well as their potential and limitation as new therapeutic approaches.

## Introduction

Influenza viruses belong to the *Orthomyxoviridae* family of single-stranded, negative sense RNA viruses with segmented RNA genomes (1, 2). There are three genera of influenza viruses including genus A, B, and C. Influenza A virus (IAV) causes significant respiratory infections in humans and global pandemics, while genus B virus can cause epidemics (but not pandemics) and genus C virus only leads to a mild disease (3).

Influenza viruses commonly cause acute respiratory infections and have posed serious threats to public health worldwide for many centuries due to their rapid and frequent mutation and recombination rate. Also, there is frequent and inevitable emergence of novel subtypes with unpredictable pathogenicity and transmissibility (4, 5). Each year, 3 to 5 million individuals experience severe influenza virus infections, with approximately 500,000 annual deaths worldwide (6–8). The clinical symptoms of acute respiratory infection by influenza viruses include high fever, body aches, headache, respiratory tract congestion, pharyngitis, and fatigue. In most cases, these symptoms are resolved in infected healthy subjects after 7–10 days. However, young children, elderly, and patients with chronic disorders such as asthma, chronic obstructive pulmonary disease (COPD), cardiovascular diseases, and diabetes are often at higher risk of developing complications such as severe bronchitis, pneumonia, or worsened medical conditions from influenza (9–14). Moreover, the severity of the disease differs between the virus subtypes (15). Although the pathogenesis and mechanisms of influenza virus infection are largely known, there is little progress in targeted intervention, either at a population level or at an individual level (to treat the cause). This is due to rapid and frequent mutations, and ineffective vaccines and antiviral drugs against influenza viruses. Furthermore, frequent antigenic changes (drift and shift) and constant emergence of drug resistant subtypes/strains also undermine the effectiveness of current anti-influenza approaches (16–20). Therefore, it is urgently required to develop more efficient approaches for prevention and treatment of the disease.

## MicroRNAs (miRNAs)

**Figure 1:**
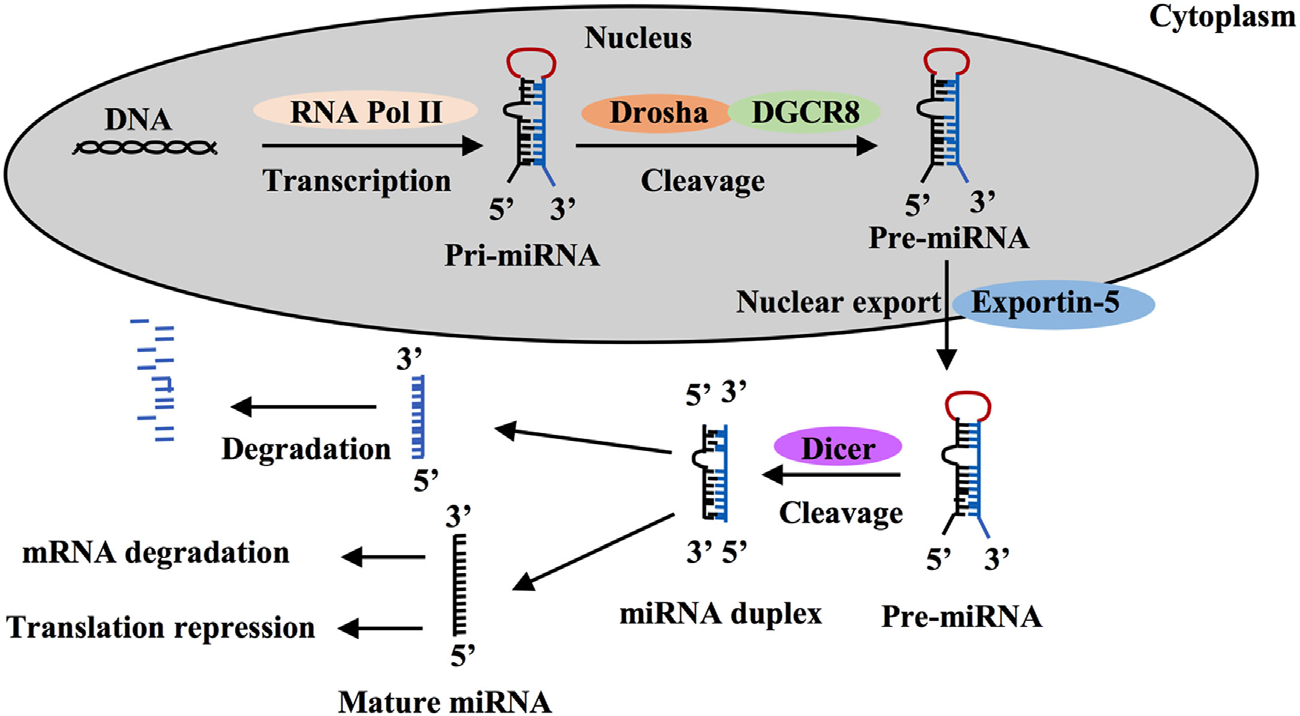
MicroRNA (miRNA) processing and function. MiRNA is first transcribed as long hairpin primary RNA (pri-miRNA) by RNA polymerase II and cleaved into hairpin precursor miRNA (pre-miRNA) by the complex Drosha-DGCR8 in nucleus. This pre-miRNA then is exported to the cytoplasm by exportin-5 protein and cleaved to two strands by endoribonuclease Dicer, one strand becomes a mature miRNA and silence target mRNAs through mRNA degradation or translation repression and the other is degraded.

MicroRNAs are a class of short non-coding single-stranded RNA sequences of about 20 bp first described two decades ago that negatively regulate gene expression in eukaryotes (21–24). These small RNAs are transcribed as long hairpin primary RNAs (pri-miRNAs) by RNA polymerase II. In nucleus, pri-miRNAs are cleaved by the microprocessor complex including Drosha ribonuclease III and the RNA-binding DGCR8 protein to form hairpin precursor miRNAs (pre-miRNAs, ~70 bp). Pre-miRNAs are exported to the cytoplasm by exportin-5 protein, belonging to the Ran-dependent nuclear transport receptor family, and are further cleaved by cytoplasmic endoribonuclease Dicer to form mature RNAs (Figure 1). Each miRNA gene has been recognized to generate two mature miRNAs that are designated as -3p miRNA and -5p miRNA (25–27). Both of them that can coexist are functional by associating with the RNA-Induced Silencing Complex. Mature miRNAs often are known to bind to 3-untranslated regions (UTRs) of target mRNAs to regulate gene expression. Most miRNA:mRNA interactions involve nucleotides 2–7 of miRNAs, a region called seed. Seed-based interactions lead to mRNA destabilization and/or translation inhibition.

Functional consequences of changed miRNA expression have been explored for diagnosis, prognosis, and severity of a wide range of diseases including infectious disease, autoimmune diseases, and cancer (28–31). Numerous investigations using anti-miRNA oligonucleotides, miRNA mimics/inhibitors, or mice deficient of a specific miRNA have demonstrated that a single miRNA can have extensive and crucial effects on the physiologic and pathological processes and that alterations in the function of miRNA can result in biological dysfunction and diseases (24, 31, 32). Despite the fact that the critical role of miRNAs in orchestrating cell differentiation, proliferation, and metabolism is well known (24, 33–35), we are only beginning to understand the contribution of these small RNAs to the innate immune responses to viral infections, to the regulation of gene expression programs, and immune cell activation (32, 34). In this review, we focus primarily on the role of miRNAs in regulating innate immune responses to IAV infections, and the potential use of miRNAs in the treatment of IAV infection.

## MicroRNAs as Diagnosis Markers

MicroRNAs are found in the intracellular niche and the extracellular fluids encapsulated in exosomes including blood plasma, serum, urine, saliva, and semen (36–38). These small molecules are also detected in body fluids independently of intracellular and exosome compartments (38). Interestingly, the differential expression of miRNAs in these compartments is linked to the development of IAV infection (29, 39–42), indicating that these small RNAs can be used as the diagnosis markers for the disease (Table 1). Evaluation of peripheral blood mononuclear cells (PBMCs) from critically ill patients with swine-origin pandemic H1N1 infection by qRT-PCR and receiver operating characteristic (ROC)/area under ROC (AUC) curve analyze has revealed that increased levels of miR-148 (>2-fold) and decreased levels of miR-31 and miR-29a (>2-fold) are valuable biomarkers for severe influenza virus infections with the AUC value ranging from 0.881 to 0.951 (39). The ROC tool is commonly used to evaluate the diagnostic accuracy of differentially expressed miRNAs for differentiating between IAV infection patients and healthy controls, and the accuracy is measured by AUC (39, 43). In another study, increased levels of miR-34c-3p (>4-fold) and decreased levels of miR-29a-3p, -30c-5p, and -181a-5p (>2-fold) have been demonstrated to be associated with the infection in the throat swab samples of H1N1-infected patients using qRT-PCR and ROC methods (43). The link between low levels of miR-29a-3p (threefold) and IAV infection has been further confirmed in the throat swabs of H1N1 infected patients using non-PCR MARS [microRNA-RNase-SPR (surface plasmon resonance)] assay (44). A similar study of peripheral blood samples from H3N2- or H1N1-infected patients using miRNA microarray and stem-loop PCR has identified that 14 miRNAs were linked to the pathogenesis of the disease (40). Among them, the levels of miR-229-5p, -335, -664, and -1260 were increased greater than twofold, while the levels of miR-18a, -26a, -30a, -34b, -185, -576-3p, -628-3p, -665, -765, and -1285 were decreased greater than fourfold. Expression of six (miR-26a, -335, -576-3p, -628-3p, -664, and -1260) of these miRNAs was confirmed in H1N1 infected A549 cells and Madin Darby Canine Kidney (MDCK) cells (40). Moreover, H7N9 has been demonstrated to cause more severe infection in humans, and to-date, this H7N9 subtype has resulted in 1,533 human infections with 592 deaths in 2017 (45). MiRNA microarray and qRT-PCR analyses using serum samples from H7N9 infected individuals showed a slightly different miRNA signature, with miR-17, -20a, -106a, and -376c being upregulated (>1.5-fold) (42). The ROC curve analysis was used to discriminate H7N9 infected patients from healthy controls for each miRNA with AUC values ranging from 0.622 to 0.988, while its value for a combination of these four miRNAs is 0.96 (42). Furthermore, miR-150 levels assessed by qRT-PCR have been found to be significantly higher (>1.5-fold) in critically ill patients than those with milder disease and healthy controls in a human study of H1N1 infection, indicating the association of this miRNA with poor disease outcome (41). Collectively, these earlier studies indicate that infection not only by different subtypes of IAVs but also by the different strains of the same IAV subtype with varying pathogenicity elicit different miRNA expression patterns in similar samples, which can be valuable diagnostic and/or prognostic markers for influenza infection and severity of the disease. Identification of these miRNAs may greatly aid the design of analytical kits for rapid and precise diagnosis of IAV subtypes of infection, and potentially to develop customized therapeutic approaches to control infection.

**Table 1:** MicroRNAs (miRNAs) as diagnosis markers.

miRNAs	Regulation^a^	Study samples	Virus strains	Reference
miR-302a	↓	Throat swab, peripheral blood mononuclear cells (PBMCs)	H1N1	(71)
miR-30c-5p	↓	Throat swabs	H1N1, H3N2	(43)
miR-34c-3p	↑	Throat swabs	H1N1, H3N2	(43)
miR-181a-5p	↓	Throat swabs	H1N1, H3N2	(43)
miR-150	↑	Serum from critically ill patients	H1N1	(41)
miR-17, -20a, -106a, -376c	↑	Serum	H7N9	(42)
miR-148a	↑	PBMCs from critically ill patients	H1N1	(39)
miR-29a -3p	↓	PBMCs from critically ill patients, throat swaps	H1N1, H3N2	(39, 43, 44)
miR-31	↓	PBMCs from critically ill patients	H1N1	(39)
miR-122, -229-5p, -1260, -335, -664, -767-5p	↑	Whole blood	H1N1	(40)
miR-1285, -185, -18, -26a, -30a, -34b, -519e, -576-3p, -826-3p, -665, -765	↓	Whole blood	H1N1	(40)

*^a^↓, downregulation; ↑, upregulation*.

## MicroRNAs Directly Target Influenza Viral RNAs

Influenza A virus consists of eight gene segments that encode for 12 viral proteins including surface glycoprotein [hemagglutinin (HA) and neuraminidase (NA)], nucleoprotein (NP), two matrix proteins (M1 and M2), three polymerase complex proteins PB1, PB2, and PA, four non-structural proteins (NS1, NS2, PA-X, and PB1-F2) (2, 46, 47). HA and NA proteins predominantly regulate virus entry and exit from host cells, and their genes are the major genetic segments for influenza antigen drift and shift by genetic mutation and reassortment to create new strains/subtypes. In contrast, other IAV viral proteins are more conservative, which is essential for IAV replication. For example, viral polymerase complex proteins (PA, PB1, PB2) and NP form a viral ribonucleoprotein (vRNP), a minimal functional unit for influenza virus replication. M1 forms a coat inside the viral envelope and binds to viral RNA. Therefore, exploration of those miRNAs that directly target those conservative viral sequences could uncover novel therapeutics to control influenza replication and propagation.

**Figure 2:**
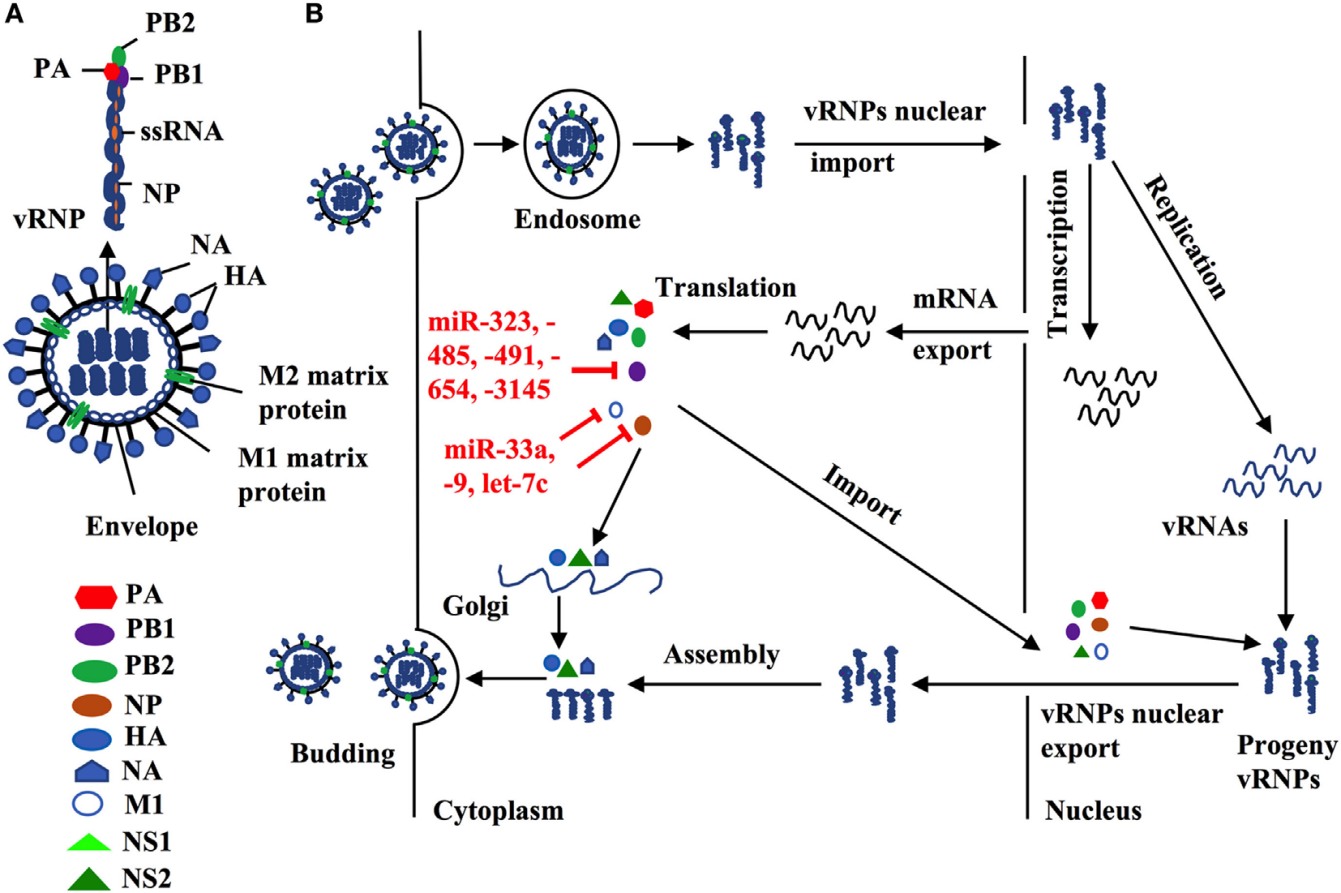
**(A)** Influenza structure: a lipid bilayer envelope containing glycoproteins M1 and M2 ion channel. Hemagglutinin and neuraminidase proteins on the outside of the envelope. Eight RNA genome segments inside the envelope encoding for three polymerase complex proteins (PB1, PB2, and PA), nucleoproteins (NPs), M1 and M2 matrix proteins, and non-structural proteins (NS1, NS2, PA-X and PB1-F2). **(B)** Cellular microRNAs (miRNAs) modulate influenza replication. Host cellular miRNAs inhibit influenza replication through targeting viral RNAs and proteins that are essential for influenza replication and translation such as PB1, M1, and nucleoprotein.

Indeed, several lines of evidence have implied the feasibility of this concept. For example, miR -323, -491, and -654 destabilize PB1 mRNA by targeting the conservative region, as demonstrated in H1N1 infected cells that are treated with plasmids carrying those miRNA mimics or inhibitors, respectively (48). A similar investigation has shown that miR-485 directly binds to a conserved site of PB1 mRNA to regulate viral replication, in H5N1-infected HEK293T cells following miR-485 mimics or inhibitor treatment (49). Furthermore, multiple miRNAs may target the same seed sequence to regulate IAV replication. Khongnomnan and colleagues, through *in silico* analysis and a luciferase reporter assay have reported that the same conservative region of PB1 mRNAs of H1N1, H5N1, or H3N2 subtypes is targeted by miR-3145 (50). Neutralization of this miRNA by using plasmid encoded anti-miRNA oligonucleotides restored the expression of PB1 mRNA and miR-3145 mimics treatment reduced PB1 expression in H5N1-, H1N1-, or H3N2-infected A549 cells (50). M1 is the most abundant protein in the IAV viral particle and regulates vRNP export, virus assembly and budding, and virus–host interactions (51, 52). Ma and colleagues have reported that let-7c precursor diminishes H1N1 replication by binding to the 3′-UTR of M1 mRNA and that let-7c inhibitor reinstates the expression of M1 protein and influenza infection in A549 cells (53). Certain microRNAs have also been shown to inhibit the expression of IAV viral proteins, not only in a direct manner but also through regulations of other host factors that affect viral replication. For example, miR-33a mimic suppressed the expression of NP and M1 proteins by directly binding to the 3′-UTR of Archain 1 (ARCN1) RNA in HEK293T, A549, and Hela cells infected with H1N1, H9N2, or H3N2, resulting in greatly decreased virus replication (54). ARCN1 is an important component of human coatomer protein complex, which regulates protein transport from the Golgi body to the endoplasmic reticulum and critically modulates influenza virus entry to host cells, viral membrane protein expression, and assembly (55, 56). Treatment with miR-33a inhibitor recovered the expression of ARCN1, NP, and M1 proteins, and thus increased H1N1, H9N2, or H3N2 replication (54). In the same study, miR-33a has also been shown to attenuate the replication of H1N1, H9N2, or H3N2 by reducing vRNP activity through an ARCN1-independent pathway in HEK293T cells (54), suggesting the multiple functions of this miRNA. A recent investigation has identified that miR-21 targets NP, PB1, PB2, PA, NA, and HA segments of H1N1, by using infected miR-21-deficient MDCK cells (28). It is promising that targeting NP segment or combination of both PA and NA segments of IAV simultaneously reduced IAV replication greater than twofold, as compared to other treatments (e.g., targeting sole segment and a combination of PA and HA) (28). Although the role of miRNAs in the pathogenesis of IAV infection should be further investigated, targeting these small viral RNAs may provide alternative approaches to reduce influenza infection by directly inhibiting expression of conserved viral proteins (e.g,. PB1, NP, or M1), regardless of the viral antigen drift and shift (Figure 2 and Table 2).

**Table 2:** MicroRNAs (miRNAs) target viral RNAs.

miRNAs	Cell types	Targets	Virus strains	Treatments	Reference
miR-485	HEK293T	PB1	H5N1	Mimic/inhibitor	(49)
miR-323, -491, -654	Madin Darby Canine Kidney (MDCK)	PB1	H1N1	Expression vectors/inhibitors	(48)
miR-3145	A549	PB1	H1N1, H5N1, H3N2	miRNA silencing vector	(50)
Let-7c	A549	M1	H1N1	Let-7c precursor/inhibitor	(53)
miR-33a	A549, HEK293T, Hela	ARCN1, viral ribonucleoprotein activity	H1N1, H9N2, H3N2	Mimic/inhibitor	(54)
miR-21	MDCK	nucleoprotein, hemagglutinin, neuraminidase	H1N1	miR-21 knockout cell	(28)

## MicroRNAs Control IAV-Induced Inflammatory Responses

Bronchial epithelial cells and pulmonary innate immune cells such as alveolar macrophages, dendritic cells, and natural killer cells provide the first line of defense against influenza infection (57, 58). Upon infection, molecular patterns of influenza viruses are recognized by host molecular pattern recognition receptors (PPRs) including retinoic acid-inducible gene I (RIG-I)-like receptors and toll-like receptors (TLRs) (59–61). These pattern-recognition receptors are important sensors that recognize infectious pathogens and drive host defense responses. Among them, TLR3 and TLR7 induces the activation of interferon (IFN) regulatory transcription factor (IRF)-3 (IRF3)/IRF7 and TIR-domain containing adaptor inducing IFN-β (TRIF)/NF-κB signaling pathways after interacting with IAV RNAs, which leads to the production of type-I (IFN-α and IFN-β) and -III (IFN-λ) IFNs and proinflammatory cytokines and chemokines (58, 62–65). RIG-I is a cytoplasmic RNA helicase that recognizes short double-stranded RNA produced during viral replication (66), and viral genomic single-stranded RNA (ssRNA) bearing 5′ phosphates (67). By binding to IAV ssRNAs, RIG-I facilitates the activation of IRF3 that directly regulate the production of type-I and -III IFNs (58, 62, 68). IAV infections are known to cause severe pro-inflammatory cytokine storm in the lung by the induction of overproduced aforementioned cytokines, which can also spread into systemic circulation, leading to severe symptoms such as leukopenia (69).

MicroRNAs have been shown to play key roles in the regulation of pro-inflammatory intracellular signaling pathways during influenza infection (32, 70–73). For example, increased expression of the IRF5 gene has been shown to be correlated with decreased levels of miR-302a (2.5-fold) in throat swab samples and PBMCs from patients with influenza infection, as compared to healthy controls (71). Treatment with miR-302a mimics decreased IRF5 expression by binding to its 3′-UTR and reduced IRF5-regulated production of IFN-β, TNFα, IL-6, IL-8, CCL2, and CCL5 in H1N1-infected PBMCs, leading to higher H1N1 viral production (71). Recently, miR-144 has been demonstrated in a mouse model to inhibit anti-IAV host responses by targeting the TNF receptor-associated factor 6 (TRAF6)/IRF7 signaling axis, which underpins type-I IFN responses against H1N1 infection (72, 74, 75). Another miRNA, miR-146a, has also been shown to directly downregulate TRAF6 in H3N2 infected human nasal epithelial cells (73). These findings suggest the importance of those miRNAs in host immunity against IAV infection by targeting TRAF6.

**Figure 3:**
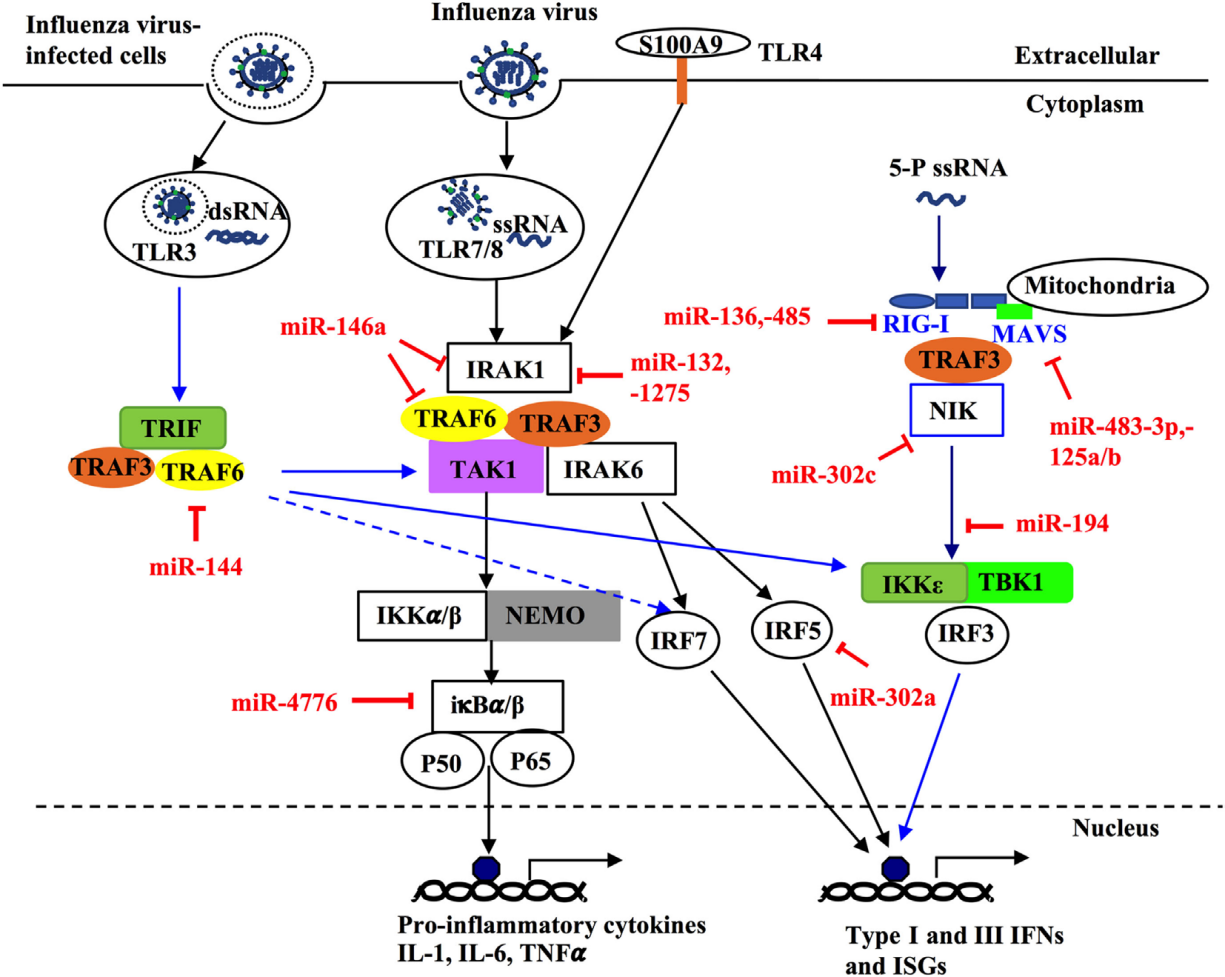
MicroRNAs (miRNAs) regulate innate host immune response against influenza infection by targeting intracellular signaling pathways. Upon infection, influenza can activate different intracellular signaling pathways such as NF-κB, RIG-like receptor (RIG-I), TNF receptor-associated factors (TRAFs), and interferon regulatory factors (IRFs) through different pattern recognition receptors including TLR3, TLR4, TLR7/8. TLR3 and TR7/8 that recognize double stranded RNA and single-stranded RNA in the endosome, respectively. The ligand for TLR4 in influenza virus is unknown; however, it is thought to be activated by the damage-associated molecular patterns molecules released in influenza virus-infected cells and trigger TLR4-MyD88-signaling pathways. Induction of these TLRs can lead to activation of NF-κB, IRF 3, 5, and 7 and induce expression of type-I and -II IFN, IFN-stimulated genes, and inflammatory genes. Some miRNAs regulate these pathways through targeting critical components such as TRAF6, IRF3, IRF5, IFR7, interleukin-1 receptor-associated kinase 1, and IκBβ. Within the infected cells, RIG-I detects the 5-triphosphorylated RNA of replicating viral genomes in cytosol and associates with mitochondrial antiviral signaling protein (MAVS) to induce pro-inflammatory cytokines and type-I IFN. miRNAs can modulate this pathway by directly targeting RIG-I, MAVS, or NF-κB-inducing kinase.

NF-κB has been shown to play important roles not only in the production of pro-inflammatory genes in response to IAV infection but also in propagation of influenza viruses (76–79). Multiple miRNAs have been identified to regulate NF-κB activation, by targeting the 3′-UTRs of its components (80–82). NF-κB inhibitor β (NFKBIB, also known as IκBβ) is a regulatory protein for NF-κB that prevents nuclear translocation of NF-κB (RelA/p65), and subsequent transcription of its target genes (83, 84). Treatment of H1N1-infected human primary bronchial epithelial cells (pBECs) with miR-4776 inhibitor led to increased expression of NFKBIB, resulting in lower viral replication (82). In contrast, miR-4776 mimic decreased the expression of NFKBIB and increased viral replication (82). Gui and colleagues have shown that H3N2 infection suppresses miR-302c expression in A549 cells (80). MiR-302c inhibitor treatment restored the expression of its targeting molecule, NF-κB-inducing kinase (NIK) that is a critical component of NF-κB pathway. MiR-302c mimic treatment prevented the nuclear translocation of NF-κB and downregulated IRF3/7 expression, leading to decreased expression of IFN-β (80). In a study of H1N1- or H3N2-infected A549 cells, miR-132, -146a, and -1275 simultaneously decreased the transcription and expression of interleukin-1 receptor-associated kinase 1, a key component of NF-κB pathway (81). These three miRNAs also target mitogen-activated kinases (MAPK) 3 that plays an important role in the activation of pro-inflammatory MAPK pathway. Those miRNAs associated with pro-inflammatory signaling pathways are delineated in Figure 3 and Table 3. Interestingly, miRNAs may contribute to virus replication by suppressing host antiviral responses. For example, Dong and colleagues recently have demonstrated with H1N1- or H3N2-infected A549 cells that miR-9 promotes IAV replication through the suppression of monocyte chemoattractant protein 1-induced protein (MCP1P1) (85). MCP1P1 is a ribonuclease that plays an important role in antiviral immune responses (86) and inhibits IAV replication by decreasing the production of M and NP proteins (85). Treatment with a miR-9 mimic greatly augmented the production of NP and M1 proteins and IAV replication by decreasing production of MCP1P1. By contrast, an inhibitor of miR-9 blocked IAV replication (85). We have also recently shown that miR-125a/b directly targets A20 deubiquitinase, an enzyme that degrades receptor interacting protein 1 and inhibits NF-κB activation (87). Infection with H3N2 or H1N1 led to increased expression of miR-125a/b, which suppressed A20 deubiquitinase production and resulted in elevated NF-κB activity and production of pro-inflammatory cytokines in pBECs of COPD patients and in *in vivo* model (87). Similarly, the association between miR-125a/b and the expression of A20 deubiquitinase was observed in the mouse model of COPD (87). Collectively, miRNAs with altered expression play important roles in IAV replication, demonstrating their potential roles of antiviral host defense responses.

**Table 3:** MicroRNAs (miRNAs) control influenza A virus-induced inflammatory and antiviral responses.

miRNAs	Cell type/models	Targets	Virus strains	Reference
miR-302a	A549	IRF5	H1N1	(71)
	Peripheral blood mononuclear cells	IFN-β, TNFα, IL-6, IL-8, CCL2, CCL5		
	Mouse			
miR-144	Mouse	TNF receptor-associated factor 6 (TRAF6)	H1N1	(72)
	Primary mouse lung epithelial cells	IRF7		
miR-146a	Human nasal epithelial cells	TRAF6	H3N2	(73)
miR-4776	Human pBECs	NFKBIB	H1N1	(82)
miR-302c	A549	NIK	H3N2	(80)
		NF-κB translocation		
		IRF3/7, IFN-β		
miR-132, -146a, -1275	A549	IRAK1	H3N2, H1N1	(81)
		MAPK 3		
miR-449b	A549	HDAC1, IFN-β	H3N2, H1N1	(102)
miR-9	A549	MCPIP1	H3N2, H1N1	(85)
miR-125a/b	pBECs of chronic obstructive pulmonary disease (COPD) patients	A20, MAVS	H3N2, H1N1	(87)
	Murine COPD model	IFN-β, p65, lung inflammation		
miR-136	A549	Retinoic acid-inducible gene I (RIG-I), IL-6, IFN-β	H5N1	(97)
miR-194	A549	FGF2, INFα, IFN-β	H1N1	(98)
	Mouse			
miR-483-3p	MLE-12	RNF5, IRF3, IFN-β, NF-κB	H1N1, H7N9, H5N1	(99)
	Mouse			
miR-132	HEK293T	P300	H5N1	(100)
		IFN-β		
miR-26a	A549, HEK293T	IFN-α/β, USP3	H1N1	(101)
miR-485	HEK293T	RIG-I	H5N1	(49)
miR-664	A549	LIF, NEK7	H7N9	(125)

*NIK, NF-κB-inducing kinase; IRF, interferon regulatory factor; IRAK1, interleukin 1 receptor-associated kinase 1; MAPK3, mitogen-activated kinase 3; HDAC, histone deacetylase; MCPIP1, monocyte chemoattractant protein 1-induced protein 1; pBECs, primary bronchial epithelial cell; RNF5, RING-finger protein 5; NFKBIB, NF-κB inhibitor β; MLE-12, mouse cell line of lung epithelial cells; USP3, ubiquitin-specific protease 3; LIF, leukemia inhibitors factor; NEK7, NIMA-related kinase 7*.

## MicroRNAs Mediate the Production of Antiviral Cytokines

Three RIG-I like receptors are crucial in antiviral host defense and consists of RIG-I, melanoma differentiation-associated protein 5 (MDA5), and laboratory of genetics and physiology 2 (LGP2) (88–90). RIG-I plays a central role in the induction of immune responses against IAV, by recognizing short RNA (67, 91). By contrast, MDA5 binds to long RNA and LGP2 acts as a positive regulator of RIG-I and MDA5 (92, 93). RIG-I recognizes viral RNA in the cytosol and interacts with the mitochondrial antiviral-signaling protein (MAVS), which is localized to the outer mitochondrial membrane (94). Aggregation of RIG-I, TRIM25, and MAVS then leads to the activation of IRF3/IRF7 by phosphorylation, which then induces the expression of type-I and -III IFNs (59, 94–96). These IFNs then bind to the respective IFN receptors on the same/neighboring cells and stimulate the expression of over 300 IFN-stimulated genes (ISGs), such as protein kinase R to limit viral replication. IAV-induced expression of miR-125a/b has also been shown to directly inhibit the expression of MAVs, leading to reduced production of type-I and III IFNs in pBECs of COPD patients and mouse model of COPD (87). Several miRNAs have been reported, either directly or indirectly, to regulate the RIG-I pathway for the antiviral response to IAV infection (97–99), this interaction is also shown in Figure 3 and Table 3. For example, H1N5 or H1N1 infection increased the expression of miR-136 and miR-194 and both have been independently shown to suppress IFN-β expression by binding to the 3′-UTR of RIG-I transcript in IAV-infected A549 cells (97, 98). Furthermore, miR-483-3p, is highly expressed in the lung during infection of mice with H1N1, H5N1, or H7N9 (99). Transfection of H1N1-, H5N1-, or H7N9-infected MLE-12, a mouse cell line of lung epithelial cells, with miR-483-3p mimic led to decreased viral replication by targeting the transcript of RING-finger protein 5, which negatively regulates RIG-I signaling pathway (99). MiR-132 has also been shown to directly targets p300, an important component of IFN-β enhanceosome, which leads to reduced induction of IFN-β (100).

New evidence has emerged on the potential role of miRNAs in IAV-induced host defense responses and in the modulation of the production of cytokines. MiR-26a significantly inhibits IAV replication by promoting the type-I IFN production and subsequent expression of ISGs in H1N1-infected A549 cells (101). IAV infection decreases the expression of histone deacetylase 1 (HDAC1) that plays an important role in the activation of type-I IFN response against IAV infection (102, 103). Furthermore, H1N1 or H3N2 infection in A549 cells induced increased levels of miR-449b (>7-fold) (104). A recent investigation has also linked the increased expression of miR-449b (>6-fold) to the decreased expression of HDAC1 and the increased expression of IFN-β in H1N1- or H3N2-induced in A549 cells (102). Interestingly, treatment of miR-449b mimics further suppressed the expression of HDAC1 and enhanced the expression of IFN-β in H1N1- or H3N2-infected A549 cells (102), suggesting the important role of this miRNA in host defense against influenza virus infections.

Interestingly, certain miRNAs have been shown to have opposing effects on regulating the antiviral response when host cells are infected at different doses of specific viruses. MiR-485 is induced by IAV infection and inhibits RIG-I pathways at low levels of IAV infection, which suppresses host antiviral responses and enhances virus replication (49). By contrast, this miRNA abates virus replication by degrading PB1 transcripts at higher levels of IAV infection (49). It is likely that varying multiplicity of infection triggers slightly different antiviral signaling pathways. Although the regulatory targets of miR-485 are currently unclear, this miRNA likely regulates those factors that are highly expressed in these conditions. Hence, the miRNA biological function should be carefully and fully examined before developing therapeutic approaches.

## Manipulation and Delivery of miRNA

Although miRNAs have been increasingly recognized in recent years as potential therapeutic targets for treating influenza infection, the successful miRNA manipulation can be difficult because of many factors such as short half-life of miRNAs, low cellular uptake and expression, pre-matured elimination by host immune cells, interruption of endogenous RNA processes and off-target effects. In particular, targeting a single miRNA may have limited success for treatment because an understanding of the molecular mechanisms underpinning many complex diseases (e.g., asthma, autoimmunity, and cancer) remains rudimentary. To elucidate the function of a miRNA family simultaneously, traditional antisense methods for a single miRNA are inadequate and laborious for targeting multiple miRNAs; to create genetically modified lab animals is even more arduous. As such, microRNA sponges have been designed to inhibit the function of a miRNA family by creating a single RNA sequence that consists of several tandem miRNA-binding sites for all the members (105). In fact, one of the unique features of miRNA function is that a family of miRNAs share an almost identical seed sequence, with often only a few nucleotides difference, although they may be expressed from different genomic loci (106–108). Therefore, targetting seed sequence may be particularly valuable using miRNA sponges to understand the pathogenesis of IAV infection and to treat the disease. A miRNA sponge could be designed to target those miRNAs that promote virus replication and IAV-induced inflammation.

How to effectively deliver miRNA sponges or mimics to manipulate host miRNAs is challenging for clinical application. For this reason, it is essential to carefully design and select candidate miRNA sequence. Non-specific responses induced by those molecules should also be examined with great caution. Any unexpected effect associated with miRNA’s target should be quantified for the safety and success of miRNA manipulation approaches. Indeed, various types of strategies have been developed to optimize the delivery methods, including chemical modification of miRNA sponge molecules or to encapsulate them with macromolecules (e.g., polyamine, polyethylenimine, and basic complexes). Furthermore, the promoters used by miRNA sponge should be strongest and most suitable for the cells of interest to achieve highest efficacy. If the targeted miRNAs are expressed in many cell types in multiple tissues, the tissue- or cell-specific promoters could be employed to achieve precise expression, which could minimize potential side effects.

Many vectors that comprise plasmid, replication-deficient virus or transposons have been developed to accomplish successful gene interference *in vivo* (109–113, 124). Among these vectors, lentiviral vectors have been widely employed in the study of normal tissue physiology and processes of disease in animal models (114, 115). For example, lentiviral vectors carrying miR-30 mimic can inhibit viral replication in H1N1-infected MDCK cells by targeting viral NP and PB1 transcripts (116). IAV itself can also be modified to express exogenous miRNAs and modulate viral replication and for the treatment of the diseases (117), as incorporation of an artificial miRNA into IVA genome does not cause viral sequence instability or interfere with viral replication (117). Langlois and colleagues have shown that recombinant H1N1 expressing artificial miR-124 does not inhibit the function of other miRNAs only with limited repression of miR-124-star target by luciferase reporter system in transfected hamster kidney cells (118), suggesting the specificity of this delivery method. Indeed, live-attenuated IAV delivery has shown great potential in the development of more efficient IAV vaccine and in the treatment of infection respiratory diseases by carrying customized artificial miRNAs (119, 120). This notion is supported by a recent study, showing that exogenous miR-155 encoded by modified X31 IAV augments IAV-specific CD8^+^ T cell response and neutralizing antibody production in a mouse model of IAV infection (120).

Adenoviral and adenovirus-associated viral (AAV) vectors are also valuable to silence candidate miRNAs *in vivo*. Indeed, recent progresses have been made in delivering a miRNA mimic to explore the therapeutic potential of these vectors in animal models (121, 122). However, adenoviruses induce off-target host defense response because they infect a wide range of cells (123). On the other hand, AAV vectors cause very few side-effects to host because they integrate at limited and defined location in the genome of transfected targets (121, 123). This unique feature of AAV vectors thus minimizes the chance of mutational insertion and induces effective gene silencing following either systemic or tissue-specific injection (121, 123). Furthermore, non-viral transfection approaches using nanoparticles and liposome have also attracted attention in the establishment of miRNA intervention. Thus, although delivery strategies are far from being optimal yet, significant progress has been achieved recently toward targeted therapy with limited off-target effects.

## Limitation

Current understanding of the roles of miRNAs in the pathogenesis of influenza is limited by a lack of sufficient characterization of the miRNA-associated molecular pathways in the context of influenza infection, IAV replication, and host immunity. Indeed, great challenge still exists in the field of the identification of miRNA targets within a living organism and in host defense processes that have many layers of molecular and cellular elements with specific spatiotemporal patterns. One of miRNA biological features is that they target multiple mRNAs; therefore, pathophysiological outcomes observed by modifying their function may correlate with subtle changes in the levels of diverse target mRNAs. Although *in vitro* assays using luciferase reporter system and miRNA mimics and inhibitors are usually employed to verify an interesting target transcript, caution should be taken to interpret the important biological function in dynamic living systems by exclusively relying on these methods. Furthermore, as miRNAs potentially act as master regulators of disease and inflammation, ill-designed manipulation of a miRNA may generate unexpected side-effects. This is particularly important when considering the observations that different species infected with different IAV strains/subtypes generate different miRNA signatures. In addition, delivery methods that limit miRNA mimics and inhibitors precisely to the infected and inflamed tissues are also needed to be further developed as it is essential for not causing any disruption to normal function of surrounding tissues.

Although many candidate miRNAs largely play suppressive roles in IAV infection, certain host miRNAs may assist viral replication. One example is that the miR-664 was highly upregulated approximately fourfold in H7N9-infected A549 cells, and treatment with miR-664 inhibitor reduced H7N9 replication (125). MiR-664 is predicted by *in silico* pathway analysis to target the 3′-UTRs of leukemia inhibitory factor (LIF) and NIMA-related kinase 7 (NEK7) whose activation leads to lower H7N9 replication (125). MiR-144 increased IAV infection by suppressing the activity of TRAF6-IRF6 axis posttranscriptionally as demonstrated in H1N1 infected miR-144 deficient mice and mouse lung epithelial cells (72). Although a specific miRNA may play either pro- or anti-IAV role, numerous investigation suggest that miRNAs have great potential as diagnostic biomarker and treatment of human diseases when considering the profound biological function regulated by these small RNAs and their extensive links to IAV infection.

## Conclusion

Based on the unique features of miRNAs, a new generation of IAV vaccine may be developed by incorporating miRNA response elements (MRE, miRNA recognition sequences) into viral genomic segments such as NP, NS, or PB1. An attempt to generate novel attenuated IAV vaccine has yielded promising results by inserting a let-7b MRE into H1N1 PB1 gene, which significantly reduced viral replication in bronchial epithelial cells (126). Although preliminary, this method to generate attenuated IAV vaccine has been proven effective in a mouse model (122).

Manipulation of miRNAs needs to be approached with caution for the reason that intervention of miRNA function may predispose to impaired immunity, cancer, or other unforeseen biological abnormalities. However, miRNA has become more and more attractive as diagnostic biomarkers and potential clinical intervention targets as effective prevention and treatments for IAV infection is poorly available. Furthermore, direct targeting of key miRNAs that underpin IAV infection may lead to new and more specific therapeutic interventions as these small RNAs are implied in regulating specific gene clusters triggered by infection (e.g., cytokine driven inflammation). It is particularly important to explore those miRNAs that can both degrade IAV RNAs and alleviate virus-induced inflammation, as they may concurrently control both virus replication and over-reactive immune responses. Understanding the role of miRNA in fundamental processes associated with IAV infection is necessary to fully characterize their potential in disease diagnosis and prognosis and ultimately for the treatment of disease.

## Author Contributions

TN and MY wrote and edited the paper. XL, ZS, AH, and PF edited the paper.

## Conflict of Interest Statement

The authors declare that the research was conducted in the absence of any commercial or financial relationships that could be construed as a potential conflict of interest.
